# Transcriptome and miRNome Analysis Provide New Insight Into Host Lipid Accumulation, Innate Immunity, and Viral Persistence in Hepatitis C Virus Infection *in vitro*

**DOI:** 10.3389/fmicb.2020.535673

**Published:** 2020-09-30

**Authors:** Chong Li, Lungen Lu, Zhongtian Qi, Yongqiang Zhu, Fengtao Su, Ping Zhao, Hui Dong

**Affiliations:** ^1^Cancer Institute, Fudan University Shanghai Cancer Center, Department of Oncology, Shanghai Medical College, Fudan University, Shanghai, China; ^2^Shanghai Key Laboratory of Pancreatic Diseases, Department of Gastroenterology, Shanghai General Hospital, Shanghai Jiao Tong University School of Medicine, Shanghai, China; ^3^Department of Microbiology, Second Military Medical University, Shanghai, China; ^4^Chinese National Human Genome Center at Shanghai, Shanghai, China

**Keywords:** hepatitis C virus, transcriptome, miRNome, host responses, lipid metabolism, miR-122

## Abstract

Hepatitis C virus (HCV)-host cell interaction during infection disturbs cellular homeostasis and culminates in pathological consequences. The processes could be first embodied in gene expression of HCV-infected cells. Here, we investigated transcriptome and miRNA expression (miRNome) alterations in HCV-infected Huh7 cells at 12, 36, and 60 h after infection to systematically explore host responses. The number of deregulated genes in the HCV-infected cells increased with infection duration. The altered biological processes at 36 h were mainly associated with stress and inflammatory response, whereas the most enriched processes at 60 h were predominantly linked to lipid metabolism. Notably, the key genes that participated in lipogenesis were downregulated, and conversely, the genes implicated in fatty acid beta-oxidation were upregulated. Reduced expression of the key genes involved in lipoprotein assembly and secretion pointed to a decreased requirement for and export of lipids, leading to lipid accumulation in HCV-infected hepatocytes. Fluctuation in the expression of host factors, innate immunity genes and transcription factors provided insight into host-directed mechanisms to control viral replication. Furthermore, miRNome presented a comprehensive expression profile of miRNAs in HCV-infected Huh7 cells. The integrated analysis of transcriptome and miRNome suggested that deregulated miR-483, miR-1303, miR-1260a, miR-27a^∗^, and miR-21^∗^ directly regulated lipid metabolical genes at 60 h. The decreased miR-122 at 60 h was indirectly involved in lipid metabolism and is expected to attenuate rampant replication of HCV and potentially contribute to viral persistence. Our results will help to gain a comprehensive understanding of the molecular mechanisms implicated in HCV-induced pathogenesis.

## Introduction

At least 71 million individuals have chronic hepatitis C caused by hepatitis C virus (HCV) (Blach et al., 2017). Chronic HCV carriers without treatment are associated with severe liver disease, including fibrosis, cirrhosis, and hepatocellular carcinoma (HCC) (Lauer and Walker, 2001).

HCV requires host factors to complete its life cycle, and virus-host factor interaction and host responses to the virus have an impact on host genes and consequently cause HCV-associated liver diseases. Gene expression changes caused by HCV have been widely reported using microarray (Blackham et al., 2010; Liu et al., 2010; Woodhouse et al., 2010) and disrupted pathways, including lipid metabolism (Blackham et al., 2010; Woodhouse et al., 2010), oxidative stress (Liu et al., 2010), and choline metabolism (Gobeil Odai et al., 2020) have been observed. These findings provide partial understanding of the viral pathogenesis, such as steatosis. However, recent evidence indicates attenuation of lipid synthesis and enhancement of fatty acid oxidation, accompanied by lipid accumulation in HCV-infected cells (Douglas et al., 2016). This finding is attributed to disrupted lipid transport (Douglas et al., 2016), which differs from the previous notion that fat accumulation is derived from increased lipogenesis stimulated by HCV (Negro, 2010). Nevertheless, molecular evidence at the transcriptomic levels for this is lacking. RNA sequencing (RNA-seq) has advantages over microarray analysis in gathering comprehensive transcriptional level information (Sultan et al., 2008; Wang et al., 2009; Ozsolak and Milos, 2011). Woodhouse et al. explored the impact of HCV infection on host cells using RNA-seq (Woodhouse et al., 2010), however, they did not investigate HCV-induced temporal expression variation, which is critical to understanding the process of HCV pathogenesis. Additionally, host factors, defense-associated genes and transcription factors whose expression patterns are critical to exploration of hepatotropism, innate immunity and gene regulation during HCV infection, respectively, have not been systematically investigated.

MicroRNAs (miRNAs) are ∼22-nucleotide-long non-coding RNAs that post-transcriptionally repress gene expression by base pairing to target mRNAs (Guo et al., 2010). Several miRNAs have been implicated in modulating critical hepatic metabolic functions; for example, miR-122 in maintaining hepatic cell phenotype (Hu et al., 2012), miR-21 in promoting lipid and glucose metabolism (Calo et al., 2016), and miR-27 in steatosis (Singaravelu et al., 2014a), as well as miR-185 and miR-130b in lipid accumulation (Singaravelu et al., 2015). miR-122 promotes HCV propagation via direct binding to two target sites in the HCV 5′ untranslated region (UTR) (Jopling et al., 2005). Another two conserved sites at the 3′ UTR (Henke et al., 2008) and coding region (Nasheri et al., 2011) of HCV RNA were reported recently. This leads to reduction of miR-122 binding to endogenous mRNA targets, functionally derepressing miR-122 targets (Luna et al., 2015). Besides miR-122, miRNAs such as let-7b, miR-199a, and miR-483 are directly or indirectly involved in HCV infection (Conrad and Niepmann, 2014; Singaravelu et al., 2014b). A global expression profile of miRNAs upon HCV infection will aid our understanding of miRNA alteration and post-transcriptional regulation after viral infection. Some dysregulated miRNAs have been reported in HCV-infected Huh7.5.1 cells using miRNA microarray analysis (Liu et al., 2010). However, smaller dynamic ranges in array compared with RNA-seq limit detection sensitivity for miRNAs expressed either at low or very high levels (Wang et al., 2009).

The human hepatoma-derived cell line (Huh7), replicating efficiently and producing infectious HCV *in vitro*, is a powerful tool for studying the viral life cycle (Wakita et al., 2005) and the most biologically representative host cell system currently available (Blackham et al., 2010). Here, we simultaneously investigated alteration of transcriptomes and miRNA expression (miRNome) in HCV-infected Huh7 cells compared with that in control cells at 12, 36, and 60 h post-infection. Our data showed intensified expression changes in both mRNAs and miRNAs with duration of infection. Transcriptomic analysis revealed decreased lipogenesis and disorder of lipoprotein metabolism at 60 h after infection, and associated abnormal lipid transport with lipid accumulation in HCV-infected cells. Systematic expression analysis provided insight into hepatotropism, host defense and transcriptional regulation upon HCV infection. Moreover, the miRNome indicated moderate alteration of miRNA expression during HCV infection. Integrative analysis of transcriptomes and miRNome identified a subset of deregulated miRNAs involved in impaired lipid homeostasis induced by HCV infection. Reduced miR-122 expression at 60 h probably plays important roles in limiting HCV rampant replication and potentially contributes to HCV persistence. These findings will advance our understanding of HCV-associated pathogenesis.

## Materials and Methods

### Production of Infectious HCV Particle

J6/JFH1 virus was produced as described previously by transfecting Huh7.5.1 with RNA transcribed from the pJ6/JFH-1 plasmid (Lindenbach et al., 2005). The plasmid was kindly provided by Dr. Rice CM. The HCV cell culture virus was multiplied by infecting Huh7.5.1 cells and the culture medium was collected 5 days after infection. The culture medium containing virus was centrifuged and filtrated through 0.45 μm pore to rid of cell debris. Afterward, the virus was concentrated by ultracentrifuge with 35,000 rpm for 4 h in 30% sucrose. Supernatant was discarded and pellet was dissolved with PBS. The final titer of the virus is about 5 × 10^5^ focus forming units/ml.

### Infection and Sample Preparation

Huh7 cells were incubated at 37°C with 5% CO_2_ in Dulbecco’s modified Eagle’s medium with 10% fetal calf serum, 100 U/ml penicillin, and 100 μg/ml streptomycin. Huh7 cells were seeded in 6-well-plates with 5 × 10^5^ cells in each well. At 12 h after cell seeding, 50 μl concentrated HCV virus was used to infect each well, and 50 μl PBS is used for mock infection (control). The cells were collected at 12, 36, and 60 h after HCV infection, respectively. There are three parallel wells for both infected and mock cells for time points 36 and 60 h. Due to low cell density at 12 h, 6 wells were used, and sample from two wells were mixed to generate three parallels.

### RNA Isolation, Quality Control, RNA-Seq Library Preparation and Sequencing

HCV-infected Huh7 cells and control were collected by centrifugation, and total RNA including miRNA was extracted using mirVana miRNA Isolation Kit (Invitrogen) according to the manufacturer’s recommendations. Total RNA was quantified and assessed for integrity using Agilent RNA 6000 Nano Kit on Bioanalyzer 2100 (Agilent Technologies). For each of 18 RNA samples, polyadenylated RNA was isolated from 1 μg of DNase I-treated (Qiagen) total RNA using Dynabeads^®^ mRNA^TM^ DIRECT Kit (Life technologies). The isolated mRNA was used to prepare RNA-seq libraries using TruSeq RNA Library Prep Kit v2 (Illumina) following manufacturer’s instruction. Libraries were sequenced on Illumina HiSeq-2500 sequencing System with read length of 100 or 150 base pairs (bps).

### Read Alignment of RNA-Seq

Between 2.9 × 10^7^ and 5.0 × 10^7^ pair-end reads for transcriptomes were obtained (Supplementary Table S1). Raw RNA-seq reads were first preprocessed using the in-house perl scripts and the sickle software (Joshi and Fass, 2011) (version, 1.200). The preprocessed reads per library were mapped against the human genome (GRCH38)^1^ using TopHat v2.0.8b^2^. Gene-level read counts were summarized with the HTSeq Python script (Anders et al., 2015) (version 0.6.0). Gene length was obtained by summing the lengths of all exons within each gene. The genes shorter than 200 bp were removed, and a total of 35,430 genes including 19,720 coding and 15,710 long non-coding genes in the Ensembl gene annotation (v.79) were measured. Genes more than 10 reads in at least three of 18 libraries were retained, resulting in a total of 18,584 detected genes including 14,235 protein-coding genes and 4,349 long non-coding RNAs, accounting for 52.5% of all genes.

### Differential Gene Expression Analysis

The Bioconductor package RUVSeq (version 1.0.0)^3^ was used to identify differentially expressed genes of HCV-infected Huh7 cells and control at the three time points. The RUVs function was used to identify differentially expressed genes in the detected genes above. A differentially expressed gene met following criteria: average expression abundance of the gene at least in a group (infected or control) is more than 10 reads per millions (RPM), false discovery rate (FDR, an adjusted *P*-value after multiple testing of Benjamini-Hochberg) (Benjamini and Hochberg, 1995) < 0.005 and fold change (FC, infected cells/control) > 1.50 (upregulation) or < -1.5 (downregulation). Unless stated otherwise, we represent fold change of gene expression (including mRNA and miRNA) by ratio of its expression abundance in HCV-infected Huh7 cells relative to control at each time point.

### Hierarchical Cluster Analysis

Hierarchical cluster analysis of differentially expressed genes between HCV-infected Huh7 cells and control at per time point was performed using the hclust function of stats package in R software^4^. Heatmaps for differentially expressed genes at any time point were plotted with heatmap.2 function in gplots package.

### Quantitative Real-Time PCR (qPCR) Validation of RNA-Seq Results

Total RNA for each sample above was reverse transcribed into cDNA using SuperScript^®^ II Reverse Transcriptase (Invitrogen). Fifty-five host genes and one HCV gene (NS3) were used to validate RNA-seq results. These genes were involved in dysregulated Gene Ontology biological processes, host factors, innate immunity and transcription factors in analysis of RNA-seq. The primers were designed online via Primer3^5^ and their sequences were presented in Supplementary Table S2. QPCRs were performed using the StepOne Plus systems (Applied Biosystems) with KAPA SYBR^®^ FAST Universal (Roche). Each gene was amplified in three replicates for each sample. Actin beta (*ACTB)* is used as endogenous control genes. For each gene at per time point, the 2^–ΔΔ*CT*^ method (Livak and Schmittgen, 2001) was applied to normalize relative gene expression change between HCV-infected cells and control.

### Gene Set Enrichment Analysis

Gene Ontology enrichment analysis of the differentially expressed genes in HCV-infected Huh7 cells was performed using the Bioconductor package goseq (see footnote) Before analysis, Gene Ontology terms associated with more than 250 genes and with very small gene sets (<15 genes) were removed because of either their too general terms difficult to interpret or more likelihood to become significant by chance alone (Backer et al., 2014). A Gene Ontology category with an adjusted *p*-value (FDR) < 0.05 and gene number ≥4 was defined as significance. Significantly enriched Gene Ontology biological processes and associated differentially expressed genes were visualized using functionalNetwork function in the Bioconductor package FGNet (Aibar et al., 2015).

### Expression Analysis of Host Factors, Immune Defense Genes and Transcription Factors in HCV-Infected Huh7 Cells

To evaluate expression of host factors during HCV infection, a total of 274 host factors were integrated from Supplementary Tables S8, S9 in literature (Li et al., 2014) and literature mining of published HCV host factors. The host factors are divided into five types based on their involvement in HCV life cycle stages, entry (E), IRES-mediated Translation (T), RNA replication (R), assembly/secretion (A), and multiple stages (M). We compiled a list of innate immune defense genes including pattern recognition receptors (PPRs) (Horner and Gale, 2013), interferon genes and interferon-stimulated genes. Of these genes, 353 interferon-stimulated genes are selected from Supplementary Tables S2–S4 in literature (Schoggins et al., 2011). And transcription factors were collected from tftargets R package^6^ which provides regulatory networks between human transcription factors and their putative targets. Here, we used datasets from ENCODE (Dunham et al., 2012), TRRUST (Han et al., 2015), and HepG2-DS7764 in Neph2012 (Neph et al., 2012) and generated a transcription factor regulation set consisting 867 transcription factors and 12,029 putative targets. The differentially expressed transcription factors and their putative targets were visualized via the “network” package in R. Host factors, interferon-stimulated genes and transcription factors are protein-coding with manual correction. To compare expression levels of these genes in detail, we classed detected genes into five categories: very low (Reads Per Kilobase per Million mapped reads (RPKM) < 0.1), low (0.1 < RPKM < 1), medium (1 < RPKM < 10), high (10 < RPKM < 100), or very high (RPKM > 100).

### Construction and Sequencing of miRNA-Seq Libraries

For each sample above, 1 μg of total RNA was used to construct small RNA library applying the Illumina TruSeq Small RNA Prep kit according to the manufacturer’s instructions. All samples were uniquely indexed and the libraries were then mixed. Sequencing of 50 cycles was performed on a Hiseq 2000 instrument (Illumina). We generated an average depth of 12.2 (range: 9.9–15.0) million reads (Supplementary Table S1), which is generally thought to be sufficient to identify moderate-to-low-abundance miRNAs and modest expression difference between samples (Lim et al., 2015).

### miRNome Analysis and Identification of Differentially Expressed miRNAs in HCV-Infected Huh7 Cells

The 3′ adapter sequence from the raw Illumina reads were first clipped using the Mapper module from the miRDeep2 (v.2.0.0.5) (Friedlander et al., 2012). Reads shorter than 18 nucleotides after clipping were discarded. The remaining reads were collapsed to identical reads in the fasta format with their frequency suffixing each identifier using the same module. The miRDeep2.pl script (Friedlander et al., 2012) was implemented to map the reads against the GRCH38 reference genome and miRNA hairpin sequences from miRBase (release v21)^7^, and summarize the counts for per sample. Mature miRNAs from the related species, *Gorilla gorilla, Pan paniscus, Pongo pygmaeus*, and *Pan troglodytes* were pooled to improve prediction performance of novel miRNAs. The miRNAs with > 10 reads in > 2 samples remained for further analysis. We obtained 681 unique mature miRNAs including 647 known and 34 novel miRNAs candidate (relative to miRBase 21). Only 25.1% of the known miRNAs were detected, suggesting cell-specific expression of miRNA species. Based on miRNA expression abundance, we also divided miRNAs into five levels, very low (RPM < 10), low (10 < RPM < 100), medium (100 < RPM < 1,000), high (1,000 < RPM < 10,000), very high (RPM > 10,000). The RUVs function in the RUVSeq package was also used to identify differentially expressed miRNAs between HCV-infected Huh7 cells and control for per time point. Differentially expressed miRNAs met FDR < 0.01, absolute (fold change) > 1.35, and average RPM in any group > 10. According to miRBase criteria (Kozomara and Griffiths-Jones, 2014), miRNAs with expression level > 10 RPM were considered as high confidence.

### QPCR Validation of miRNome Results

Total RNA for 6 samples at 60 h was reverse transcribed using All-in-One^TM^ miRNA qRT-PCR Detection Kit 2.0 (GeneCopoeia) following manufacturer’s manual. miR-122, miR-483, miR-1303, and miR-1260a were used to validate miRNome data. Primer sequences (Product No. of GeneCopoeia) are HmiRQP0056 (miR-122), HmiRQP0100 (miR-1260a), HmiRQP0150 (miR-1303), and HmiRQP0517 (miR-483). QPCRs were performed using the 7300 Plus systems (Applied Biosystems). Each miRNA was amplified in three replicates for each sample. Human sapiens snRNA U6 (HmiRQP9001, GeneCopoeia) was used as endogenous control. For each miRNA, the 2^–ΔΔ*CT*^ method (Livak and Schmittgen, 2001) was applied to normalize relative gene expression change between HCV-infected cells and control.

### Regulatory Analysis of Differentially Expressed Genes Targeted by miRNAs

TargetScan (Agarwal et al., 2015) (Release 7.0) was used to predict targets of differentially expressed miRNAs. In addition, miRTarBase (Chou et al., 2018) (Release 6.1), a database collecting experimentally validated microRNA–target interactions, was also included to construct target sets of the differentially expressed miRNAs. Based on the principle that miRNAs and their predicted targets have inverse expression change in mammalian cells (Guo et al., 2010), we matched differentially expressed genes to target sets of the differentially expressed miRNAs to identify actual regulatory relationship between mRNAs and miRNA in HCV-infected Huh7 cells. The predicted targets and differentially expressed genes at 60 h were separately uploaded to DAVID^8^ to conduct enrichment analysis of Gene Ontology biological processes and KEGG pathways. These predicted targets were further matched to host factors, interferon-stimulated genes and transcription factors to generate their relationship with the differentially expressed miRNAs. Regulatory networks between differentially expressed miRNAs and their targeted host factors, interferon-stimulated genes and transcription factors were plotted via the “network” package in R (see footnote).

## Results

### Intensified Alteration in Transcriptome of HCV-Infected Huh7 Cells Over Time

**FIGURE 1:**
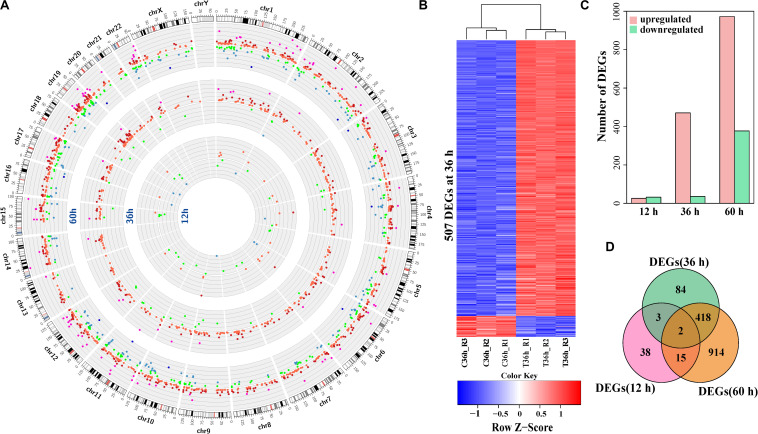
Expression alterations in HCV-infected Huh7 cells compared to control. **(A)** A Circos plot of the differentially expressed genes (DEGs) between HCV-infected Huh7 cells and control at the three time points after infection. The outermost ring shows the human chromosomes. From inside to out, the other three rings present the differentially expressed genes at 12, 36, and 60 h, respectively. Each dot on any ring represents a differentially expressed gene at that time point. Green and red indicate downregulation and upregulation of differentially expressed genes in HCV-infected Huh7 cells compared to control, respectively. Intensity of fold-change (FC, HCV-infected cells/control) is shown in different colors. Green, blue and deep blue indicate –2 < FC < –1.5, –2 < FC < –4 and FC < –4, respectively, red, deep red and purple correspond 1.5 < FC < 2, 2 < FC < 4, and FC > 4, respectively. **(B)** Heatmap of the differentially expressed genes at 36 h. Each row represents a DEG. Red indicates higher expression and blue indicates lower expression in HCV-infected Huh7 cells. **(C)** Composition of up-regulated and down-regulated differentially expressed genes at 12, 36, and 60 h. **(D)** Venn plot of the differentially expressed genes in HCV-infected Huh7 cells at the three time points.

To explore the impact of HCV infection on host cells, we performed RNA-seq of HCV-infected Huh7 cells at 12, 36, and 60 h after infection. During infection, HCV RNA levels at 12 h were low and dramatically increased but comparable at 36 and 60 h (Supplementary Figure S1A). Of the genes detected by RNA-seq, 58 (0.31%), 507 (2.73%), and 1,349 (7.26%) were differentially expressed genes between infected and control cells at 12, 36, and 60 h, respectively (Figure 1A and Supplementary Table S3). Hierarchical clustering of the differentially expressed genes demonstrated manifestly different expression patterns between infected and control cells (Figure 1B and Supplementary Figure S1B). Quantitative real-time PCR (qPCR) validated expression changes detected by RNA-seq with a Pearson’s correlation coefficient of 0.95 (*p*-value: 9.15 × 10^–39^, Supplementary Figure S1C). Most (92.9%) of the differentially expressed genes at 36 h were upregulated in HCV-infected cells (Figure 1C). Only *KLHL24* and *C10orf12* were dysregulated across all the time points (Figure 1D), and these shared differentially expressed genes between different times showed expression fluctuation (Supplementary Figures S1D–F). These data showed aggravated effects of HCV infection on Huh7 cells with duration of infection.

### Persistent Infection of HCV Disturbed Lipid Metabolism in Infected Huh7 Cells

**FIGURE 2:**
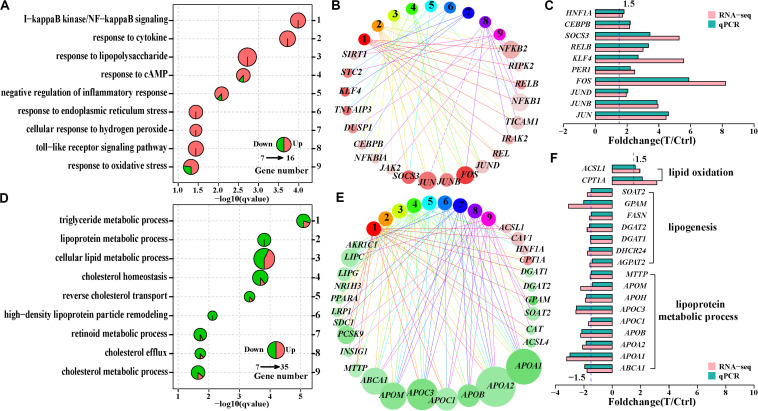
Comparison of the significantly enriched Gene ontology (GO) biological processes at 36 and 60 h. **(A)** Nine significantly enriched GO biological processes associated with inflammatory status at 36 h. **(D)** Nine significantly enriched GO biological processes linked to lipid and lipoprotein metabolism at 60 h. One pie represents a significantly enriched GO biological process, and pie size reflects differentially expressed gene number in that GO biological process. The sector area on a pie indicates ratio of upregulated (red) or downregulated (green) genes to the differentially expressed genes in that GO biological process. **(B,E)** Functional network of GO biological process and the differentially expressed genes based on the GO biological process in **(A,D)**, respectively. Each circle with number corresponds an enriched GO biological process in **(A,D)**, respectively, and each circle with gene symbol represents a differentially expressed gene. The GO biological process and its corresponding genes are linked through edges. Red and green indicates upregulation and downregulation of genes, respectively. The color depth of a circle (a differentially expressed gene) reflects intensity of fold change and the circle size is in proportion to the number of the enriched GO biological process which the gene is involved in **(C,F)**. qPCR validation of the genes involved in multiple GO biological processes in **(B,E)**, respectively.

To assess the biological significance of the differentially expressed genes, we carried out enrichment analysis of Gene Ontology terms of biological processes. The two Gene Ontology biological processes with small gene numbers were significantly enriched at 12 h, while 23 and 30 Gene Ontology biological processes at 36 and 60 h, respectively, were significantly altered after HCV infection (Supplementary Table S4). Although there were 420 overlapped differentially expressed genes between 36 and 60 h, only circadian rhythm (GO:0007623), reverse cholesterol transport (GO:0043691), and positive regulation of cell differentiation (GO:0045597) were shared (Supplementary Figures S2A,B). This suggested temporal difference in cellular function with duration of infection. The most significantly altered Gene Ontology biological processes was I-κB kinase/nuclear factor (NF)-κB signaling (GO:0007249) at 36 h, other Gene Ontology biological processes included responses to type of stimulus, including cytokine (GO:0034097), cAMP (GO:0032496), lipopolysaccharide (GO:0035994), and endoplasmic reticulum stress (GO:0035914), implying inflammatory conditions in Huh7 cells at 36 h (Figure 2A). Functional network analysis showed that *NF-κB* (*NFKB2*, *REL*, and *RELB*) and *AP1* (*JUN*, *JUNB*, *JUND*, and *FOS*) families, as well as *SOSC3*, *KLF4*, and *CEBPB* were involved in the multiple Gene Ontology biological processes (Figure 2B), probably playing a critical role in inflammatory status at 36 h. Their expression changes were validated using qPCR (Figure 2C). In contrast, the most significantly enriched Gene Ontology biological processes at 60 h were linked to cellular lipid metabolic processes, including metabolism of triglycerides, cholesterol and lipoproteins (Figure 2D and Supplementary Figure S2B). Of note, most of the genes implicated in these Gene Ontology biological processes, such as *FASN*, *DGAT1*, *DGAT2*, *GPAM*, *SOAT2*, *DHCR24*, and *AGPAT2*, were downregulated (Figures 2E,F). These genes were critical for lipid synthesis, implying reduced lipogenesis in Huh7 cells at 60 h. Another subset of genes associated with lipoprotein assembly and transport, including *ABCA1*, *MTTP*, and apolipoprotein genes (*APOA1*, *APOA2*, *APOB*, *APOC1*, *APOC3*, *APOH*, and *APOM*) were also downregulated (Figures 2E,F). Additionally, they participated in multiple Gene Ontology biological processes at 60 h (Figure 2E), suggesting their abnormal expression could contribute to dysfunction of the infected cells. These findings indicated that HCV persistence could cause disequilibrium of lipid metabolism in the infected Huh7 cells.

### Effects of HCV Infection on Expression of Host Factors Involved in the HCV Life Cycle

**FIGURE 3:**
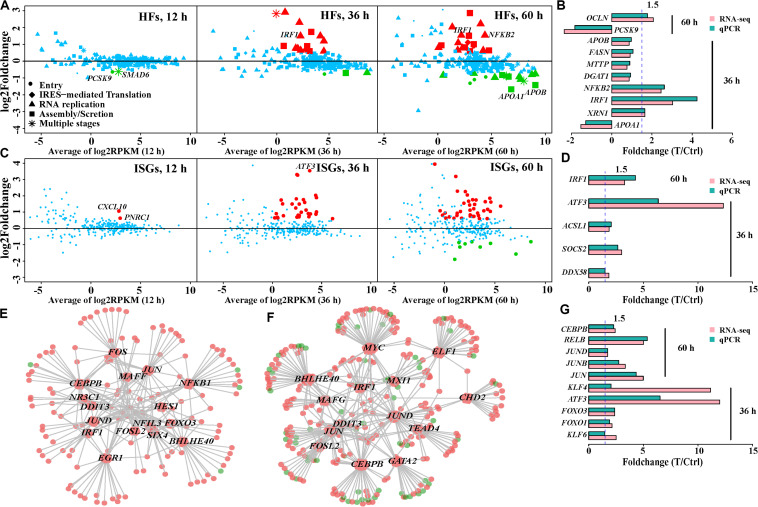
Expression patterns of host factors (HFs), interferon-stimulated genes (ISGs), and regulation of transcription factors (TFs) in HCV-infected Huh7 cells over infection time. **(A)** Expression variation of the host factors in HCV-infected Huh7 cells at the three time points. Dots, rhombi, triangles, squares, and asterisks represent the different involvement of host factors in the HCV life cycle. “Others” indicates the host factors involved in two steps. Upregulated and downregulated differentially expressed genes are displayed in red and green, respectively, and genes with no expression change are shown in blue (the same below). **(B,D,G)** qPCR validation of partial host factors, interferon-stimulated genes and transcription factors, respectively. **(C)** Expression pattern of the interferon-stimulated genes. **(E,F)** Regulatory network of the DE transcription factors and their putative targets (differentially expressed genes) at 36 and 60 h, respectively. Each hexagon represents a differentially expressed transcription factor and each circle indicates the putative target. Size of a hexagon is proportional to number of genes which this transcription factor regulates. The links (gray lines) between hexagons and circles displays regulation relationship. Upregulation and downregulation are displayed in red and green, respectively. Transcription factors with more than 10 putative targets, named as hub transcription factors, were plotted.

Host factors are required for the HCV life cycle, while interaction of HCV and host factors can regulate expression of host factors and cause functional disorder of host cells. We compiled a list of 274 host factors to evaluate expression of host factors during HCV infection. These host factors were simply divided into five types based on their involvement in the viral life cycle. Only *PCSK9* and *SMAD6* showed expression variation at 12 h (Figure 3A). This is consistent with prior reports that HCV infection negatively regulates *PCSK9* expression (Syed et al., 2014), and its downregulation facilitates initial interaction between HCV and Huh7 cells. These host factor**s** were simply divided into five types based on their involvement in the viral life cycle. Eighteen and 35 host factors were dysregulated at 36 and 60 h, respectively (Figure 3A and Supplementary Table S5), while some canonical entry factors (Scheel and Rice, 2013) including *CD81*, *CLDN1*, *CLDN9*, as well as *EGFR* and *EPHA2* (Lupberger et al., 2011), were not transcriptionally varied. Except for *CLDN9* with low expression, the other four genes were highly or very highly expressed, especially *CLDN1* with > 300 RPKM. This indicated that baseline expression of these factors in Huh7 cells may be sufficient for HCV entry. In fact, > 50% of the detected host factors were highly expressed (RPKM > 10) without expression alteration during infection. This implied that additional expression changes of these host factors were not necessary for HCV infection.

Given the proviral or antiviral effects of these host factors, we further examined their regulatory mechanisms to distinguish the actual roles of these differentially expressed host factors upon HCV infection. Based on their fold-change in expression and action on HCV, we observed two types of regulatory mechanism of host factor**s**. Type 1 referred to the mechanism by which the host factors were regulated to limit HCV replication. In this regard, host factors were upregulated with antiviral effect or downregulated with proviral effect. They accounted for 66.6 and 71.4% of the differentially expressed host factor**s** at 36 and 60 h, respectively (Supplementary Table S5). This type included upregulated *RIPK2*, *IRF1*, *NFKB2*, *RELB*, *XRN1*, and *SOCS3* (antiviral) at 36 h, as well as downregulated *SDC1*, *DGAT1*, *HSPA8*, and *APOA1* (proviral) at 60 h (Figure 3B). These genes were also involved in host inflammatory status and lipid metabolic processes. Type 2 showed an opposite mode of action to facilitate viral hepatotropism and propagation; for example, upregulated proviral *PLA2G4C*, *NUAK2*, and *OCLN*, and downregulated antiviral *PCSK9* at 60 h (Figure 3B).

### Expression Spectrum of Innate Immune Defense Genes During HCV Infection

PPRs include Toll-like receptor (*TLR*) family, *MDA5*, and *RIG-I* (*DDX58*). *TLR* family members had low or no expression in this study, suggesting their less involvement in HCV immunity. *RIG-I* was transcribed with medium expression and upregulated at 60 h after infection, while *MDA5* (*IFIH1*) showed low mRNA levels. Their downstream effect could induce transcription of antiviral type I and III interferons (IFNs) (Horner and Gale, 2013), nevertheless, we failed to detect any expression of type I, II, and III IFNs (22 genes) in Huh7 cells. We further investigated the expression spectrum of 353 interferon-stimulated genes associated with the type I IFN response. Despite over 70% of 294 detected interferon-stimulated genes with RPKM > 1, 30, and 49 differentially expressed interferon-stimulated genes were observed at 36 and 60 h, respectively (Figure 3C and Supplementary Table S6). These included *IRF1*, *GTPBP2*, *MAP3K14*, and *CYP1B1*, which displayed high levels of inhibition of HCV infection in a previous study (Schoggins et al., 2011). Expression levels of *ATF3* in HCV-infected cells were 10-fold higher than those in control cells at 36 and 60 h. The increased expression of *ATF3* was similarly observed in other HCV-infected Huh7.5.1 (Liu et al., 2010) and Huh7.5 cells (Woodhouse et al., 2010). Expression changes of partial interferon-stimulated genes are shown in Figure 3D.

### Expression Alteration of Transcription Factors and Their Regulatory Roles During HCV Infection

Aberrant expression of host genes during HCV infection led us to investigate regulatory roles of transcription factors. We observed two, 77 and 104 dysregulated transcription factors of the 867 compiled transcription factors (Supplementary Table S7 and Supplementary Figure S3) at 12, 36, and 60 h, respectively, partially contributing to more disturbed genes with duration of infection. Most (>87%) of these differentially expressed transcription factors were transcriptionally enhanced in HCV-infected cells at 36 and 60 h (Supplementary Figure S3). The most dramatically upregulated transcription factors included *ATF3*, *KLF4*, *JUN*, *KLF10*, *JUNB*, and *FOSL2*. A subset of 61 overlapped transcription factors with the same change tendency at 36 and 60 h indicated their persistent involvement in HCV infection. These included *JUN*, *JUNB*, and *JUND* in the *AP1* family; *NFKB2*, *RELB*, and *REL* in the *NFKB* family; as well as *KLF4*, 6, 7, 9, 10, 11, and 15 in the *KLF* family.

Of the integrated transcription factors regulatory network set, we observed 58 and 79 altered transcription factors, regulating 153 and 372 differentially expressed genes at 36 and 60 h, respectively. Two sets of the top-most connected transcription factors (hubs) accounted for 83.0% (127/153) and 73.1% (272/372) of the regulated differentially expressed genes, respectively (Figures 3E,F). About 50% of differentially expressed interferon-stimulated genes at 36 and 60 h were putatively targeted by these transcription factors, suggesting that changes in interferon-stimulated gene expression could not be induced by IFN pathway. Eight shared hub transcription factors by the two time points comprised *CEBPB*, *JUN*, *JUND*, *FOSL2*, *IRF1*, *BHLHE40*, *DDIT3*, and *SIX4* (Figure 3G), suggesting their sustaining regulatory roles.

### The miRNome of HCV-Infected Huh7 Cells Over Time

**FIGURE 4:**
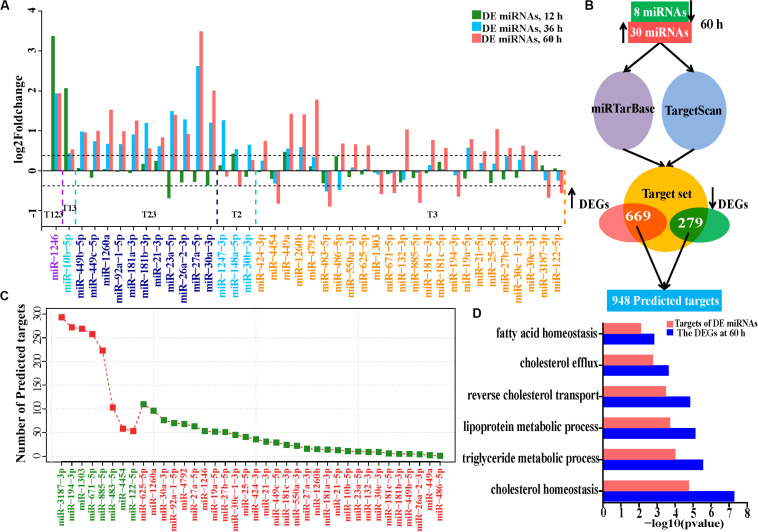
Expression alteration and regulation of miRNAs in HCV-infected Huh7 cells. **(A)** The differentially expressed miRNAs at the three time points. miR-1246 is up-regulated at 12, 36, and 60 h and indicated by T123, and so on, for the other DE miRNAs. **(B)** Schematic representation of predicted targets regulated by the DE miRNAs at 60 h. **(C)** Number of predicted targets regulated by the differentially expressed miRNAs at 60 h. **(D)** Comparison of the enriched GO biological processes between all differentially expressed genes and the predicted targets by the 38 miRNAs at 60 h. GO biological processes associated with lipid metabolism were presented.

Given the roles of miRNAs in HCV infection and their function (Jopling et al., 2005; Nasheri et al., 2011; Singaravelu et al., 2014a) in post-transcriptional regulation of gene expression (Guo et al., 2010), we further conducted miRNA-seq of the 18 samples to evaluate the effects of HCV infection on miRNA expression and involvement of miRNA in dysfunction of HCV-infected Huh7 cells. Most (75.6%) of the aligned miRNA-seq reads were accounted for by miRNAs and the remaining RNAs were represented by other classes of small RNAs (Supplementary Figure S4A). Seventy-five highly and very highly expressed miRNAs (Supplementary Figure S4B) dominated miRNA expression abundance (94.2%), which is consistent with previous findings (Fehniger et al., 2010). Expression distribution of these miRNAs and the most abundant miRNAs in Huh7 cells were shown in Supplementary Figures S4C,D. Although miR-122 is expressed at the most abundant level in the liver (Hu et al., 2012), it makes up only 2.05% of all miRNA expression abundance. Dramatic reduction of miR-122 in Huh7 cells compared with human primary hepatocytes was similarly observed previously (Chang et al., 2004). We identified 2, 15, and 37 differentially expressed miRNAs in infected cells at 12, 36, and 60 h, respectively (Figure 4A and Supplementary Figure S5). Of the differentially expressed miRNAs, 50–87.5% were expressed with low and medium abundance. Notably, only a few miRNAs with very high expression abundance were observed with modest deregulation until 60 h, such as miR-21 and miR-122 (Supplementary Figure S5). Although several miRNAs, including miR-1246, miR-10b-5p, miR-27a-5p (miR-27a^∗^), and miR-30a-3p, presented with intensive upregulation (fold change > 3.5), most of the differentially expressed miRNAs showed moderate expression variation (1.35–2.0-fold reduction or increase, Figure 4A).

### Integrative Analysis of miRNome and Transcriptome

We combined TargetScan (Agarwal et al., 2015) and miRTarBase (Chou et al., 2018) to construct a target set of DE miRNAs. We matched differentially expressed genes to the target set and obtained a median of 1,792 putative targets for each differentially expressed miRNA (Supplementary Figures S6A,B). On average, only 2.56% of the combined target sets of each miRNA showed the expected expression changes (Supplementary Figure S6B). 9.8% (5/51), 5.3% (26/490), and 72.1% (948/1,314) of the protein-coding differentially expressed genes were putatively regulated by the corresponding miRNAs at 12, 36, and 60 h, respectively, implying that miRNA regulation is limited in HCV-infected cells at 12 and 36 h.

**FIGURE 5:**
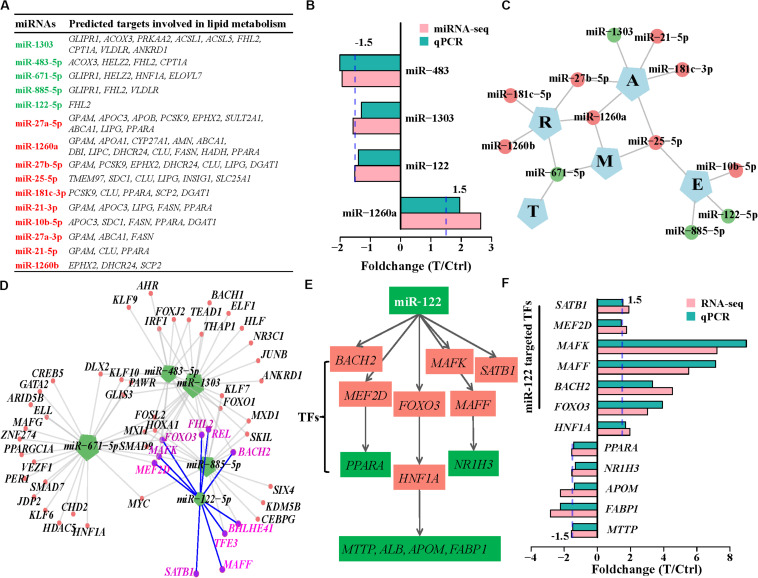
miRNA regulation in lipid metabolism induced by HCV. **(A)** The 15 highly expressed DE miRNAs and their targets involved in lipid metabolism. Upregulated and downregulated miRNAs are labeled by red and green, respectively. **(B)** Expression changes of the deregulated miRNAs detected by miRNome were validated by qPCR. **(C)** Regulatory networks between the downregulated miRNAs and the five categories of host factors. E, entry; T, IRES-mediated translation; R, RNA-replication; A, assembly/secretion; and M, multiple states. **(D)** Downregulated miRNAs and their transcription factor targets. Each edge between genes and miRNAs indicates regulation relation. miR-122-targeted transcription factors are highlighted with purple. **(E)** miR-122 indirectly regulates lipid metabolism via transcription factors. **(F)** Expression variation of transcription factors regulated by miR-122 and their downstream targets were validated by qPCR.

We further investigated the regulatory roles in biological processes of 38 differentially expressed miRNAs. Eight downregulated miRNAs regulated 669 genes, while 30 upregulated miRNAs repressed 279 targets (Figures 4B,C and Supplementary Table S8). These 948 targets also enriched in lipid metabolism (Gene Ontology biological processes, Figure 4D and KEGG pathways, Supplementary Figure S7A), suggesting that these differentially expressed miRNAs directly regulate lipid metabolism during HCV infection. We then presented a subset of the 15 top differentially expressed miRNAs based on their expression levels (RPM > 100) and their predictive targets implicated in lipid metabolism (Figure 5A). These data showed that upregulated miR-1260a, miR-21-3p (miR-21^∗^), and miR-27a^∗^, as well as downregulated miR-483 and miR-1303 were likely to exert direct regulatory effects on lipid metabolic disorder in HCV-infected cells (Figure 5A). The targets of the three upregulated miRNAs included *ABCA1*, *APOA1*, *APOB*, *APOC3*, *FASN*, *GPAM*, *LIPC*, *LIPG*, *SCD*, *SPTLC3*, and *DHCR24.* Most of them were validated by qPCR (Figure 2F and Supplementary Figure S7B). miR-483 and miR-1303 were linked with upregulation of *VLDLR*, *ACSL5*, *ACOX3*, and *CPT1A*. Notably, the targets underwent cross-regulatory action, for example, *FASN* was depressed by miR-1260a, miR-21^∗^, miR-27a-3p, and miR-10b-5p (Figure 5A). Expression changes of miR-122, miR-483, miR-1303, and miR-1260a in miRNome were validated by qPCR (Pearson’s cor > 0.99 with *p* = 0.0049, Figure 5B).

We next assessed the regulatory roles of the 15 miRNAs in host factors, and interferon-stimulated genes. Based on predictive host factors of the 15 miRNAs, miR-122, miR-10b, and miR-885 were involved in HCV entry (E), miR-1303, miR-21, and miR-181-3p regulated HCV assembly/secretion (A), as well as miR-181c and miR-1260b participated in regulation of HCV replication(R). However, other miRNAs including miR-671, miR-27b-5p, miR-1260a, and miR-25 exerted functions in multiple types of host factors and could not be clearly clustered as RNA-seq did (Figure 5C and Supplementary Table S8). In analysis of interferon-stimulated genes regulated by miRNAs, miR-1303, miR-671, and miR-885 targeted more interferon-stimulated genes than other miRNAs did, implying their more roles in regulation of interferon-stimulated gene expression (Supplementary Figure 7C and Supplementary Table S8).

Although miR-122 is critical to cholesterol and fatty acid metabolism in the liver (Esau et al., 2006); nevertheless, of the 53 deregulated putative targets of miR-122, only *FHL2* is directly associated with lipid metabolism (Figure 5A). Most of the targets were linked with amino acid transport, possibly with assistance of downregulated miR-483-5p and miR-885-5p (Supplementary Figure S8). It was not known whether miR-122 exerted indirect effects on expression of genes involved in lipid metabolism through the downstream activities of miRNA-targeted transcription factors. We further analyzed regulatory roles of the 15 miRNAs in transcription factors. Of the 53 miR-122 targets, *BACH2*, *MAFF*, *MAFK*, *MEF2D*, *REL*, *FOXO3*, *BHLHE41*, and *SATB1* belonged to the integrated transcription factor set (Figure 5D). *MAFF*, *MEF2D*, and *FOXO3* regulated *NR1H3*, *PPARA*, and *HNF1A*, respectively. Inhibition of PPARA promotes hepatocellular triglyceride accumulation (Rakic et al., 2006; Singaravelu et al., 2014a). *MTTP*, *ALB*, *APOM*, and *FABP1* were targets of transcription factor HNF1A and involved in lipid metabolism (Figure 5E). Expression changes of the transcription factors and their targets were confirmed by qPCR (Figure 5F). These provided possible evidence that miR-122 acted indirectly in lipid metabolism through regulating transcription factors and their targets.

## Discussion

Attack and defense between HCV and its host disturb the normal physiological function of host cells. The process could be first reflected in deregulation of gene expression. Our data demonstrated aggravated gene expression disorder in HCV-infected Huh7 cells and dynamic alterations of cellular physiology with infection duration.

Coupled with a dramatic increase in HCV RNA levels, the HCV-induced inflammatory milieu was observed in infected cells at 36 h, while persistent HCV infection caused lipid metabolic imbalance at 60 h. Downregulated genes associated with lipid synthesis comprised *FASN*, *DGAT1*, *DGAT2*, *SOAT2*, *GPAM*, *DHCR24*, and *AGPAT2* at 60 h, which points to attenuated lipogenesis in HCV-infected cells. For instance, FASN catalyzes synthesis of long-chain saturated fatty acids (Jeon et al., 2012), and DGAT1 and DGAT2 function in triglyceride biosynthesis and are essential in lipid droplet (LD) biogenesis (Yen et al., 2008). SOAT2 is responsible for generation of cholesterol esters (CE) (Zhang et al., 2014). Moreover, decreased lipogenesis appears to be concomitant with enhanced fatty acid beta-oxidation, for upregulated *ACSL1*, *ACSL5*, and *CPT1A*. ACSL1 and ACSL5 convert long chain fatty acids (LCFAs) into fatty acyl-CoA (Soupene and Kuypers, 2008), and the latter are transported by CPT1A across the mitochondrial inner membrane for oxidation of LCFAs (Stefanovic-Racic et al., 2008). The combined effects could be not conducive to lipid accumulation. Nevertheless, it is well documented that HCV infection is frequently associated with steatosis (Jarmay et al., 2005; Negro, 2010), characterized by cytoplasmic accumulation of LDs. Our results seem to be contradictory with these observations at first glance. However, attenuated lipid synthesis and increased mitochondrial fatty acid oxidation were similarly observed in HCV-infected Huh7.5 cells (Douglas et al., 2016), and increase in cellular lipids in HCV-infected Huh7.5 cells was reported (Woodhouse et al., 2010; Douglas et al., 2016). Besides, significantly decreased low density lipoproteins (LDL) and cholesterol in serum of HCV patients relative to the uninfected control were found (Corey et al., 2009). These findings implied altered lipid transport in HCV-infected cells. High-density lipoprotein (HDL), very low-density lipoprotein (VLDL), and LDL are mainly responsible for transport of cellular triglyceride and cholesterol ester, and apolipoproteins constitute protein components of the lipoproteins. Upload of lipids to apolipoproteins is required for formation of lipid-rich lipoproteins. For example, lipidation of APOA1 mediated by ABCA1 is a rate-limiting step in HDL biogenesis (Lee and Parks, 2005). Hepatic VLDL assembly requires upload of lipids to nascent apoB via MTTP, and its secretion is dependent on sufficient amounts of *apoB*, *MTTP* and various lipids (Davis, 1999). Indeed, abnormal lipoprotein metabolism and HDL particle remodeling were enriched. Strikingly, a subset of apolipoproteins including *APOA1*, *APOA2*, *APOB*, *APOC1*, *APOC3*, *APOH*, and *APOM* implicated in multiple processes of lipid and lipoprotein metabolism were downregulated in infected cells at 60 h. *ABCA1* and *MTTP* were also downregulated. *MTTP* downregulation is linked to hepatic steatosis in mice (Tsai et al., 2012). Reduced expression of these genes could partially relieve the contradiction between reduced lipogenesis and increased lipid accumulation in HCV-infected cells. Decreases in hepatocellular apolipoprotein levels at 60 h after HCV infection likely attenuate assembly and secretion of lipoproteins. This is believed to lower the requirement for and secretion of triglyceride and cholesterol, leading to accumulation of the two species in infected hepatic cells. Clinical evidence supporting this includes lower serum cholesterol and triglyceride levels in HCV patients (Jarmay et al., 2005; Dai et al., 2008) and hypobetalipoproteinemia (Serfaty et al., 2001). Collectively, the net effects of these deregulated genes at 60 h could contribute to accumulation of cellular lipids.

Close to 50% of the host factors in Huh7 cells were expressed at high levels, strongly supporting the hepatotropism of HCV. Especially, the major entry factors *CD81*, *EGFR*, *LDLR*, *SCARB1*, *CLDN1*, and *NPC1L1* show high expression, which probably suffices for HCV entry. Based on expression abundance of host factors, our data clearly indicated the real host dependency of HCV. Host factors with low or very low expression were less likely associated with HCV life cycle; for example, *CLDN9* for HCV entry. HCV appeared to be capable of mediating expression of host factors to facilitate its replication; for instance, type 2 host factors promoted viral propagation. In contrast, > 66% of type 1 host factors including *XRN1*, *IRF1*, and *APOA1* at 36 and 60 h could counteract HCV infection in favor of host cells. Upregulated *XRN1* that degrades replicating HCV RNA from the 5′ end in infected cells (Li et al., 2013) could improve antiviral effects. Given that HCV RNA levels were high and comparable at 36 and 60 h, protective expression of type 1 host factors was not sufficient for host cells to resolve HCV infection, but they could mitigate HCV propagation.

Host cells rely on PPRs to recognize specific features of HCV and trigger antiviral signaling (Horner and Gale, 2013). RIG-I can activate downstream NF-κB and IRF3 to induce synthesis of pro-inflammatory cytokines and IFN production (Horner and Gale, 2013). Surprisingly, no mRNA expression of IFNs was detected in our study. This implied failure to activate IFN signaling in HCV-infected Huh7 cells unless their mRNAs exist too transiently to be detected by RNA-seq. Given that constitutively expressed *IRF3* (RPKM > 10) is critical in activation of IFN response (Loo and Gale, 2011), it is likely that translocation of *IRF3* into the nuclei, where activated IRF3 regulates IFNB expression, is prevented. Similarly, limited IRF3 activation is observed in chronic HCV-infected hepatocytes (Lau et al., 2008). This prevention is attributed to cleavage of MAVS from intracellular membranes by the HCV NS3/4A protease (Loo et al., 2006). Failed induction of IFNs is also likely linked to ATF3 and KLF4. ATF3 suppresses type I IFN expression in mouse and human cells (Cai et al., 2014). Enhanced transcription and secretion of INF-γ was demonstrated in *ATF3*null NK cells (Rosenberger et al., 2008). Overexpression of *KLF4* inhibited virus-induced activation of *IFNB* via inhibition of recruitment of *IRF3* to *IFNB* promoter (Luo et al., 2016). Dramatic elevation of ATF3 and *KLF4* expression at 36 and 60 h probably strengthens their role in transcriptional repression of IFNs and thereby acting as a positive regulator for HCV replication. Despite absence of IFNs, a subset of interferon-stimulated genes was induced by HCV-infection at 36 and 60 h, indicating that an alternative regulatory mechanism of interferon-stimulated gene induction independent of IFN pathway existed. Upregulated *IRF1* appeared to play a compensatory role in this regulation, for *IRF1* overexpression transcriptionally activates a set of interferon-stimulated genes overlapping those induced by type I IFN (Schoggins et al., 2011). For example, *DDX58* and *SOSC2* are targets of *IRF1* in transcription factor analysis. Considering high HCV RNA abundance at 36 and 60 h, combined effects of the induced interferon-stimulated genes did not mount an effective immune response to eradicate HCV; nevertheless, these interferon-stimulated genes could act cooperatively to retard viral replication. Altogether, antiviral action of these interferon-stimulated genes was limited, and spontaneous HCV clearance could require collaborative effects of more interferon-stimulated genes through activating IFN signaling.

Global gene expression variation in HCV-infected cells covers a broad range of deregulated transcription factors. *NF-κB*, *JUN*, and *KLF* families were noteworthy and members of them were regarded as hub transcription factors in analysis of the regulatory networks (Figures 3E,F). Upregulated *RELB*, *NFKB2*, *JUN*, *JUND*, and *KLF4*, *6*, *7*, *10*, and *11* in HCV-infected Huh7.5 cells are also observed (Woodhouse et al., 2010), suggesting regulatory similarity upon HCV infection. The three *NF-κB* and *AP1* members are involved in responses to cytokines and lipopolysaccharide, and *I-κB* kinase/*NF-κB* signaling, probably leading to a persistently inflammatory state in HCV-infected cells at 36 h. Besides, the *AP1* family is directly implicated in regulation of cell proliferation and differentiation. The AP1 family regulates *REL*, *RELB*, and *ATF3* in bioinformatics analysis, indicating that the family probably acted as a positive and upstream modulator of inflammatory signaling. As transcriptional regulators, *KLF* family members exert diverse and essential effects in cell proliferation, differentiation and inflammation (McConnell and Yang, 2010). The upregulated *KLF* members during HCV infection could disturb related biological processes. In fact, KLF4 participates in multiple Gene Ontology biological processes including cellular regulation of inflammatory response, proliferation and hydrogen peroxide. KLF6 is a pro-fibrogenic transcription factors and associated with fibrosis in non-alcoholic fatty liver disease (Miele et al., 2008).

Given the moderate fold changes in DE miRNAs in HCV-infected cells, the extent to which miRNAs were influenced by HCV was modest overall. However, genes targeted by differentially expressed miRNAs are significantly associated with lipid metabolism, and involved in regulation of expression of host factors, interferon-stimulated genes and transcription factors, implying that miRNAs play critical roles in deregulating cellular function during HCV infection. Our data support that miR-1260a, miR-21^∗^, and miR-27a^∗^, as well as miR-483 and miR-1303 are likely to exert directly regulatory effects in lipid metabolism. miR-27a^∗^ was dramatically elevated at 36 and 60 h, although increased miR-27 expression by HCV has been reported (Singaravelu et al., 2014a). *APOB*, *APOC3*, *ABCA1*, and *PCSK9* are candidate targets of miRNA-27a^∗^ and linked with cholesterol homeostasis. miR-21/miR-21^∗^ in stressed hepatocytes are upregulated and promote lipid metabolic disorders (Calo et al., 2016) and both were enhanced at 60 h in our study. Although miR-122 is involved in lipid metabolism (Esau et al., 2006), our analysis revealed indirect regulation of miR-122 in this process. The remaining deregulated miRNAs, including miR-30-3p and miR-34-5p/449-5p families at 60 h showed low expression levels or a significant but mild increase. This makes it unlikely that they have a substantial biological effect in HCV-infected cells, because lowly expressed miRNAs have little regulatory capacity (Brown et al., 2007). Taken together, not only miR-122, but also other deregulated miRNAs cooperatively tune gene expression in Huh7 cells, especially genes associated with lipid metabolic processes.

miR-122 binds to two tandem sites close to the 5′ end of the HCV genome, conferring viral stability and promoting HCV replication (Jopling et al., 2005; Li et al., 2013). Besides, HCV exploits its interaction with miR-122 to protect its genome from degradation of the 5′ exonuclease XRN1 (Li et al., 2013). Surprisingly, miR-122 was downregulated at 60 h. Hepatic miR-122 was also found to be downregulated in chronic HCV patients (Butt et al., 2016). Downregulated miR-122 in concert with upregulated *XRN1* probably decays excess HCV RNA, which is in accordance with comparable HCV RNA levels between 36 and 60 h after HCV infection. Here, miR-122 appeared to act as a cellular “brake” to limit rampant viral spread. Of note, miR-122 levels revealed about 50% reduction in the infected cells relative to control cells at 60 h. The significant but modest downregulation is conducive to viral persistence, whereas lower miR-122 levels could cause unexpected side effects such as impaired lipid homeostasis of host cells. The miR-122 has multiple roles in regulating lipid metabolism (Esau et al., 2006; Tsai et al., 2012). Given that HCV RNA functionally sequesters miR-122 and reduces its binding to endogenous targets (Luna et al., 2015), miR-122 repression further aggravates the process. The important gene expression alteration at 60 h after HCV infection resembled that in previous studies of miR-122 inhibition (Esau et al., 2006) or genetic deletion (Tsai et al., 2012). For example, *FASN*, *SCD*, *MTTP*, and *KLF6*, which are the key genes regulating lipid metabolism, fibrosis and steatosis (Miele et al., 2008; Negro, 2010; Jeon et al., 2012; Lyn et al., 2014), showed consistent change in expression in our study and in two previous studies (Esau et al., 2006; Tsai et al., 2012). Additionally, inhibition of miR-122 in mice increased hepatic fatty-acid oxidation and decreased fatty-acid synthesis (Esau et al., 2006), which was similar to Gene Ontology term enrichment analysis at 60 h. The miR-122 repression has been reported in liver cirrhosis and HCC (Tsai et al., 2012), which are linked causally to chronic HCV infection (Lauer and Walker, 2001). The coincidence implied that miR-122 downregulation was likely to be tightly implicated in the pathological manifestations induced by persistent HCV infection. Although the underlying mechanism by which host cells (for active defense) or HCV itself (for persistence) partially repress miR-122 is not clear, some evidence suggests that *NR1D1* could contribute to the decrease in miR-122, because Nr1d1 transcriptionally repressed miR-122 in mice (Gatfield et al., 2009). Moreover, *NR1D1* was significantly upregulated in HCV-infected cells at 60 h.

**FIGURE 6:**
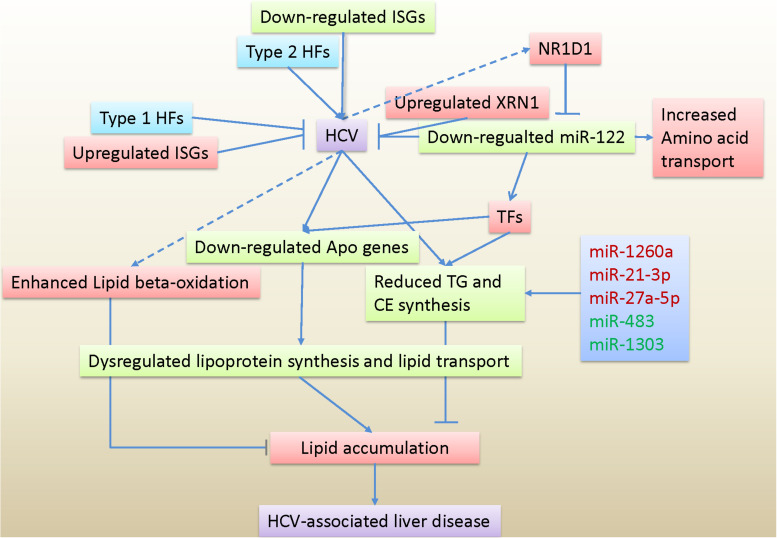
Hypothetical model of host responses to HCV infection based on transcriptome and miRNome data. TG, triglyceride; CE, cholesterol esters; Apo genes, apolipoprotein genes; HFs, Host factors; ISGs, interferon-stimulated genes; TFs, Transcription factors.

In summary, our data provided evidence at the transcriptional levels for aberrant lipid transport caused by disrupted lipoprotein metabolism, which primarily contributed to lipid accumulation in HCV-infected hepatocytes. Expression analysis of host factors, innate immune genes and transcription factors advanced our understanding of host responses to HCV and transcription regulation in infected cells. Unlike miR-122, deregulated miR-1260a, miR-21^∗^, and miR-27a^∗^, as well as miR-483 and miR-1303 were directly involved in lipid metabolism. The miR-122 repression could be a potential mechanism to control rampant replication of HCV and achieve viral persistence, probably with cooperation of type 1 host factors and upregulated interferon-stimulated genes. Based on our data, a hypothetical model of host responses to HCV infection is presented in Figure 6.

## Data Availability Statement

The datasets presented in this study can be found in online repositories. The names of the repository/repositories and accession number(s) can be found below: the National Center for Biotechnology Information, Sequence Read Archive (SRA) Accession Number: SRA accession: PRJNA559203.

## Author Contributions

HD and PZ designed and conceptualized the experiments. CL analyzed, validated, and visualized the experimental data, and wrote the original draft. ZQ and YZ supported the experiments and data analysis. LL and FS provided resources and funding. HD, PZ, and FS reviewed and edited the manuscript. All authors contributed to the article and approved the submitted version.

## Conflict of Interest

The authors declare that the research was conducted in the absence of any commercial or financial relationships that could be construed as a potential conflict of interest.

## Footnotes

1 https://asia.ensembl.org/info/data/ftp/index.html

2 http://ccb.jhu.edu/software/tophat/index.shtml

3 http://www.bioconductor.org

4 https://www.r-project.org/

5 http://bioinfo.ut.ee/primer3-0.4.0/

6 https://github.com/slowkow/tftargets

7 http://www.mirbase.org/

8 https://david.ncifcrf.gov/tools.jsp

## Supplementary Material

Supplementary Figure 1 Temporal alteration in HCV RNA abundance and gene expression in HCV-infected Huh7 cells. **(A)** HCV RNA abundance in HCV-infected Huh7 cells and control at the three time points. Data are presented as the mean ± SD (standard deviation). HCV RNA in control was not detected and their abundance was set to 0. **(B)** Heatmap of the differentially expressed genes at 60 h. Red indicates higher expression and blue indicates lower expression in HCV-infected Huh7 cells. **(C)** Comparison of fold change between RNAseq and qPCR method. A blue triangle represents a gene. **(D–F)** Fold change fluctuation of the shared differentially expressed genes between any two time points. Number of the shared differentially expressed genes was labeled on the top-left of the plot.

Supplementary Figure 2 Significantly enriched Gene Ontology (GO) biological processes of the differentially expressed genes at 36 and 60 h. **(A)** The 23 enriched GO biological processes based on the 490 differentially expressed genes at 36 h. The GO biological processes labeled by red are associated with inflammatory status. **(B)** The 30 enriched GO biological processes based on the 1,314 differentially expressed genes at 60 h. The GO biological processes labeled by green are involved in lipid and lipoprotein metabolism. One pie represents a significantly enriched GO biological process, and its size reflects differentially expressed gene number in that GO biological process. The gene number of the smallest and biggest pies is labeled on each plot. The sector area on a pie indicates ratio of upregulated (or downregulated) genes to the differentially expressed genes in that GO biological process. Red and green indicate up and downregulated genes, respectively. The three GO biological processes enriched both at 36 and 60 h were highlighted by red.

Supplementary Figure 3 Expression pattern of the transcription factors in HCV-infected Huh7 cells at the three time points. Upregulated and downregulated differentially expressed genes are displayed in red and green, respectively, and the genes with no expression change are showed in blue.

Supplementary Figure 4 Expression profile of miRNAs in HCV-infected Huh7 cells. **(A)** Ratio of the small RNAs to total expression abundance in Huh7 cells. Each bar represents average expression proportion of more than two replicates at a time point. The right bars at the dashed red line are proportional to the right *y*-axis. **(B)** Characteristic of miRNA expression levels in Huh7 cells. The miRNA expression levels are mean of 17 analyzed samples. **(C)** Histogram of average miRNA expression abundance of 17 samples. **(D)** The 25 highest expressed miRNAs with > 5,000 RPM in nine HCV-infected Huh7 cells and eight control. The order in expression abundance of the miRNAs is slightly different between infected cells and control.

Supplementary Figure 5 miRNA expression variation in HCV-infected Huh7 cells and control over infection time. A total of 42 DE miRNAs at the three time points were presented. *Y*-axis indicates miRNA expression abundance with reads per million (RPM). The purple-labeled character on each plot shows whether the miRNA is dysregulated at that time, for example, miR-1246 was significantly deregulated at 12, 36, and 60 h, and labeled by T123. The other labels refer to the legend. Data are shown as the mean ± SD.

Supplementary Figure 6 Number of and percentage of differentially expressed targets in the integrated targets of the 42 miRNAs. **(A)** Predicted targets of the differentially expressed miRNAs. Darkorange rectangles indicate number of predicted (TargetScan) and curated targets (miRBase) of the miRNAs. Dodgerblue triangles indicates percentage of the differentially expressed targets in predicted and curated targets and the percentage corresponds to the right *y*-axis. **(B)** Boxplot of predicted targets in **(A)**.

Supplementary Figure 7 Pathways and interferon-stimulated genes regulated by miRNAs. **(A)** Comparison of the enriched KEGG pathways between all differentially expressed genes and the predicted targets by the differentially expressed miRNAs at 60 h. The pathways involved in lipid metabolisms are exhibited. **(B)** qPCR validation of gene expression changes targeted by miRNAs. **(C)** Interferon-stimulated genes regulated by downregulated miRNAs. Each edge between miRNAs and interferon-stimulated gene represents a predictive regulation.

Supplementary Figure 8 Deregulated miRNAs and their putative involvement in the enriched Gene Ontology (GO) biological processes at 60 h. X-axis represents deregulated miRNAs and y-axis indicates enriched GO biological processes. Gene number in one GO biological process regulated by one miRNA is labeled in box. Expression levels and alteration of the miRNAs were presented using bars and colored fold changes, respectively. The Number of differentially expressed genes targeted by miRNAs in a GO biological process and all differentially expressed genes belonging to that GO biological processes were highlighted by orange at the right.

Supplementary Table 1 Sequencing depth for RNA-seq and miRNA-seq in HCV-infected Huh7 cells and control.

Supplementary Table 2 Primer sequences for qPCR validation.

Supplementary Table 3 The differentially expressed genes in HCV-infected Huh7 cells at 12, 36, and 60 h.

Supplementary Table 4 The significantly enriched Gene Ontology (GO) biological processes of the differentially expressed genes.

Supplementary Table 5 The differentially expressed (DE) host factors in HCV-infected Huh7 cells at 12, 36, and 60 h.

Supplementary Table 6 The differentially expressed (DE) interferon-stimulated genes (ISGs) in HCV-infected Huh7 cells at 12, 36, and 60 h.

Supplementary Table 7 The differentially expressed (DE) transcription factors (TFs) in HCV-infected Huh7 cells.

Supplementary Table 8 Predicted targets regulated by 38 differentially expressed (DE) miRNA at 60 h.
