# Supplementary figures and images for: Transcriptome and miRNome Analysis Provide New Insight Into Host Lipid Accumulation, Innate Immunity, and Viral Persistence in Hepatitis C Virus Infection in vitro
Source: Front Microbiol. 2020 Sep 30;11:535673. doi: 10.3389/fmicb.2020.535673 (PMC7555709; doi:10.3389/fmicb.2020.535673)

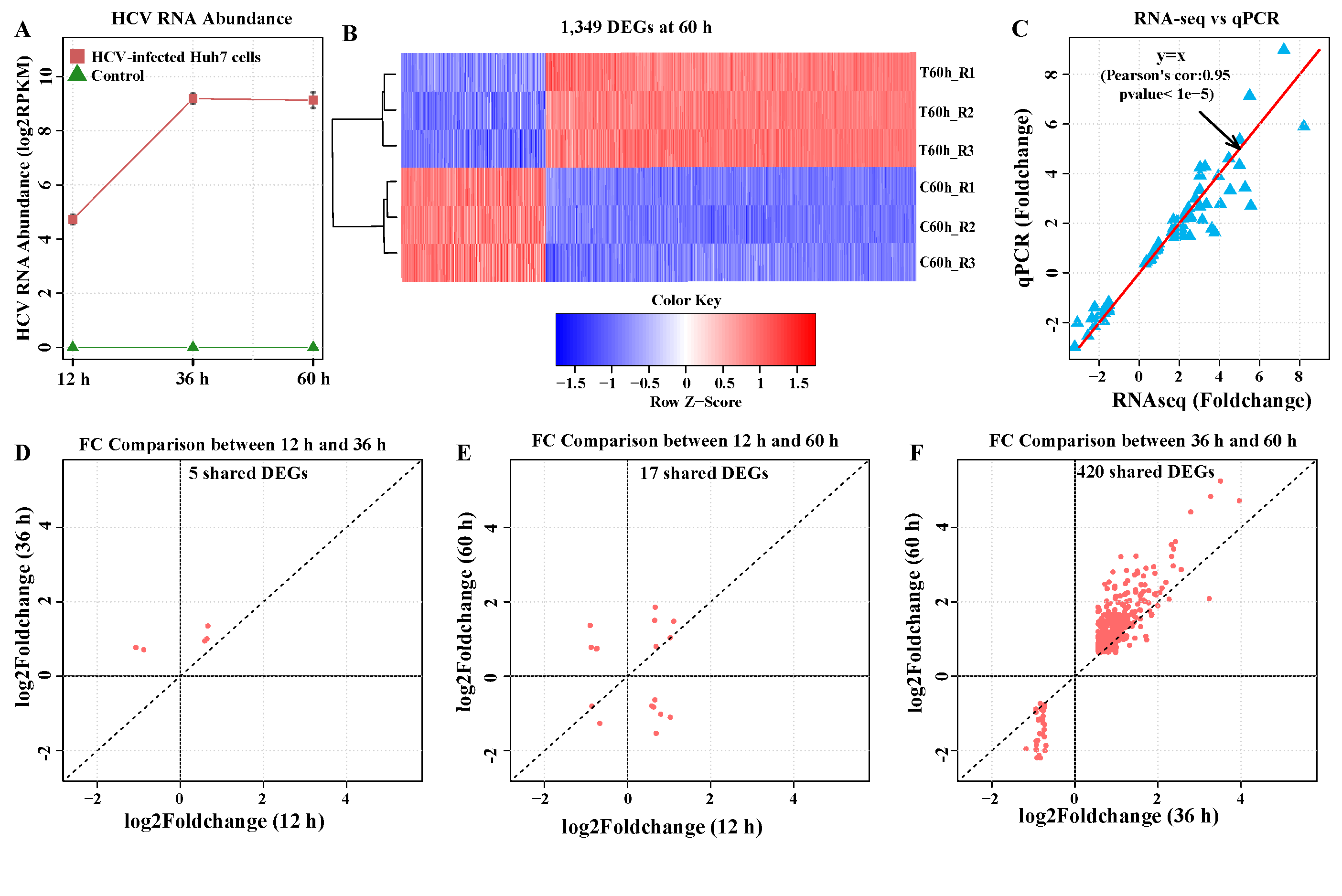

Supplement: Supplementary Figure 1 — Temporal alteration in HCV RNA abundance and gene expression in HCV-infected Huh7 cells. (A) HCV RNA abundance in HCV-infected Huh7 cells and control at the three time points. Data are presented as the mean ± SD (standard deviation). HCV RNA in control was not detected and their abundance was set to 0. (B) Heatmap of the differentially expressed genes at 60 h. Red indicates higher expression and blue indicates lower expression in HCV-infected Huh7 cells. (C) Comparison of fold change between RNAseq and qPCR method. A blue triangle represents a gene. (D–F) Fold change fluctuation of the shared differentially expressed genes between any two time points. Number of the shared differentially expressed genes was labeled on the top-left of the plot. [file Image_1.TIF]

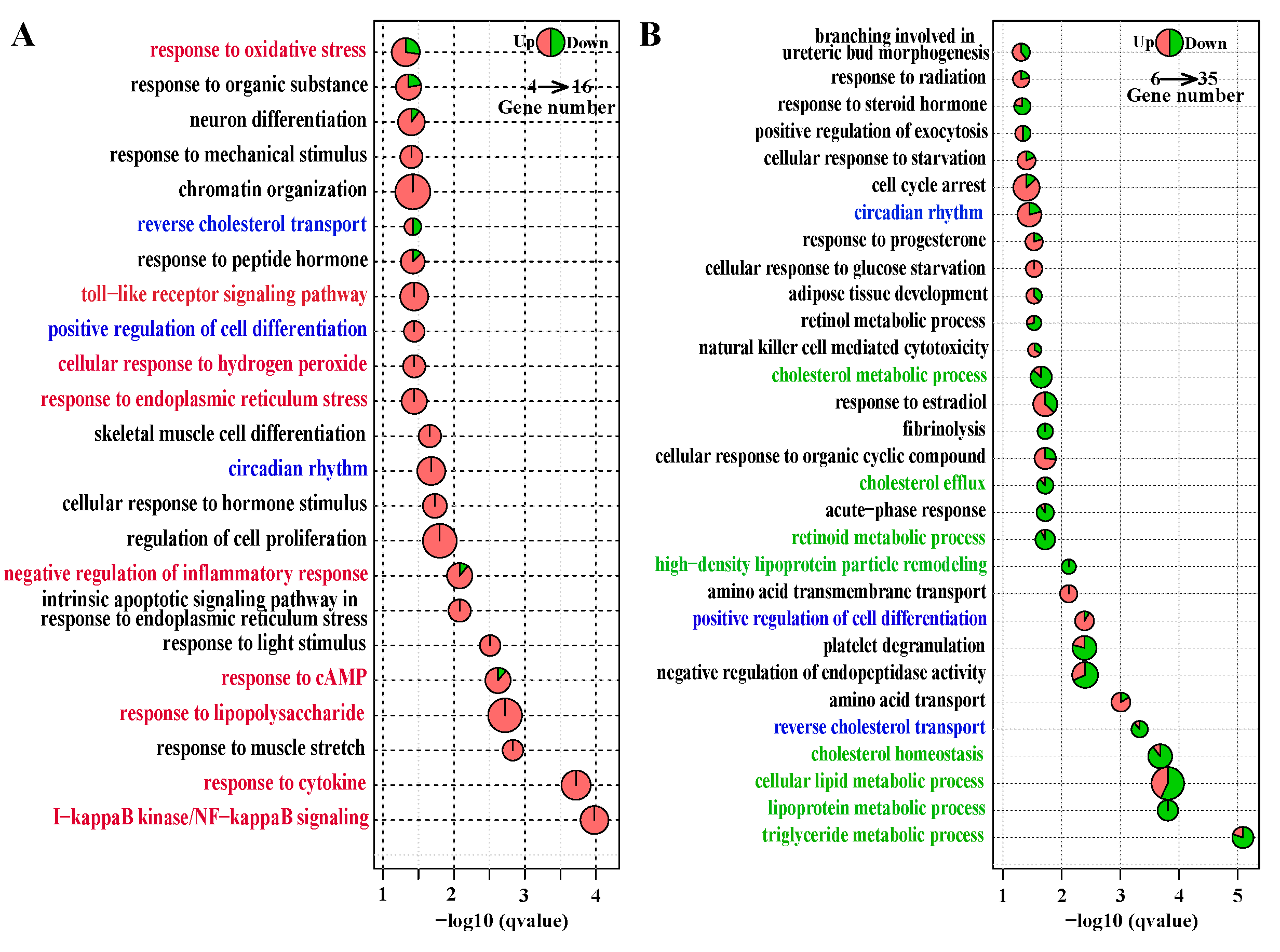

Supplement: Supplementary Figure 2 — Significantly enriched Gene Ontology (GO) biological processes of the differentially expressed genes at 36 and 60 h. (A) The 23 enriched GO biological processes based on the 490 differentially expressed genes at 36 h. The GO biological processes labeled by red are associated with inflammatory status. (B) The 30 enriched GO biological processes based on the 1,314 differentially expressed genes at 60 h. The GO biological processes labeled by green are involved in lipid and lipoprotein metabolism. One pie represents a significantly enriched GO biological process, and its size reflects differentially expressed gene number in that GO biological process. The gene number of the smallest and biggest pies is labeled on each plot. The sector area on a pie indicates ratio of upregulated (or downregulated) genes to the differentially expressed genes in that GO biological process. Red and green indicate up and downregulated genes, respectively. The three GO biological processes enriched both at 36 and 60 h were highlighted by red. [file Image_2.TIF]

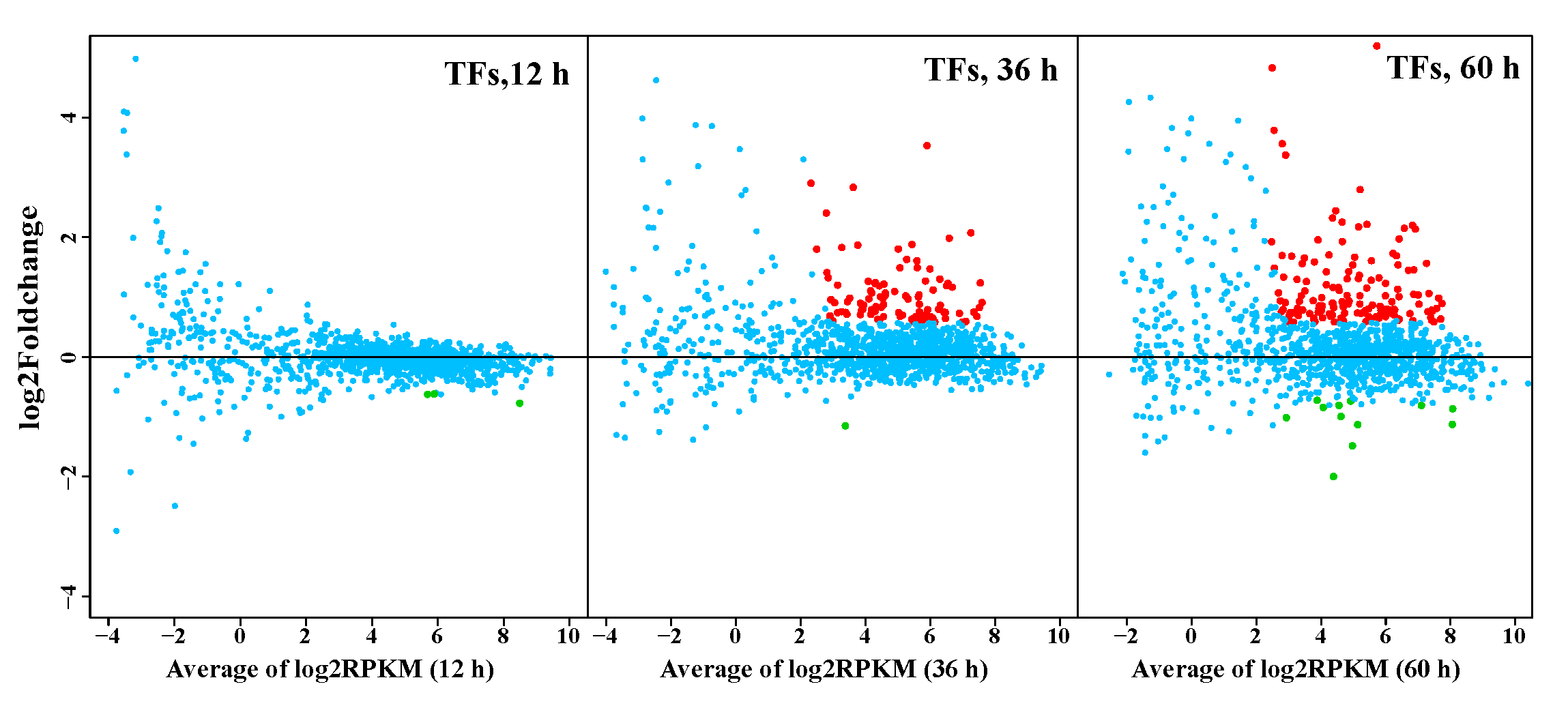

Supplement: Supplementary Figure 3 — Expression pattern of the transcription factors in HCV-infected Huh7 cells at the three time points. Upregulated and downregulated differentially expressed genes are displayed in red and green, respectively, and the genes with no expression change are showed in blue. [file Image_3.TIF]

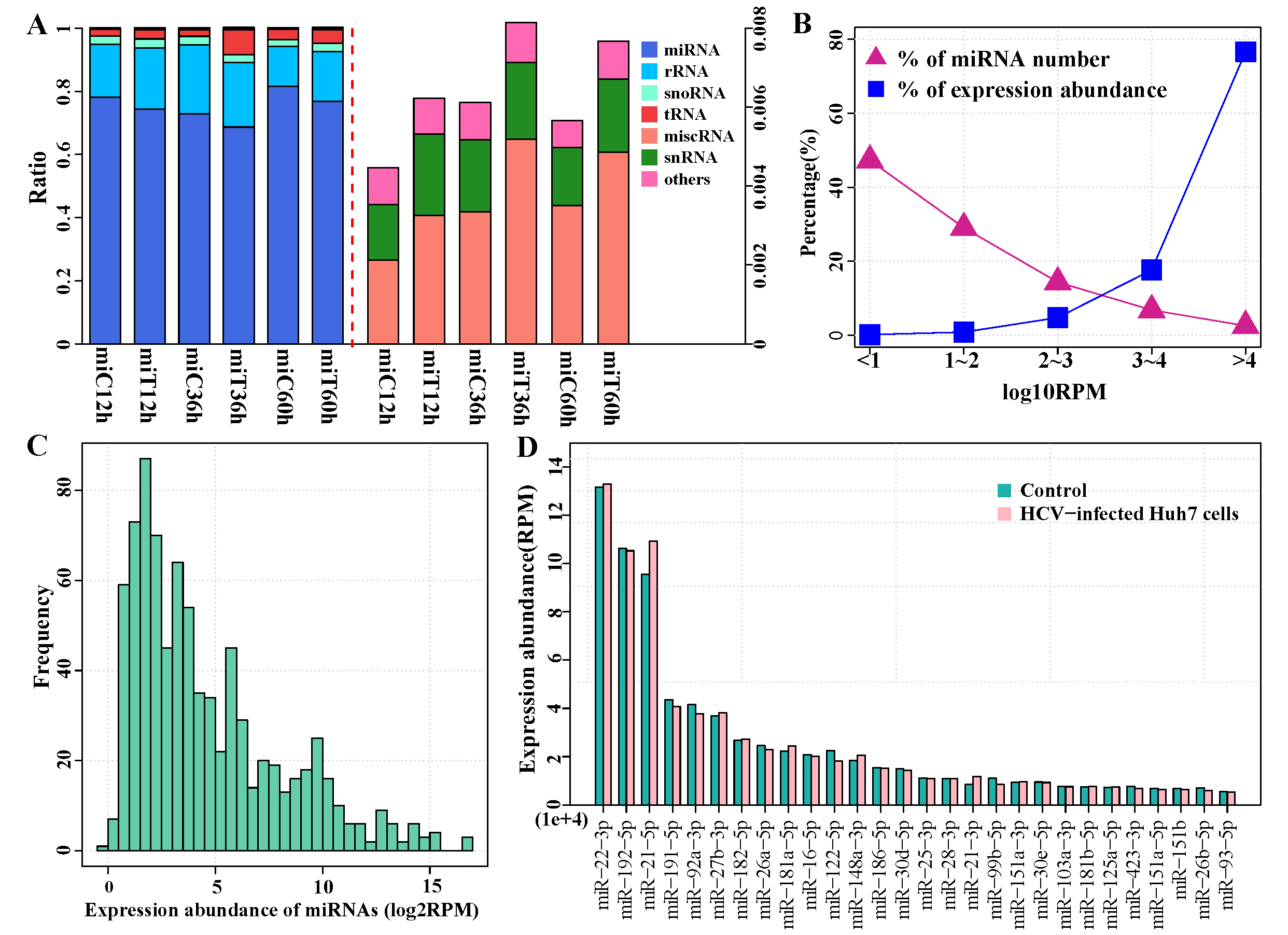

Supplement: Supplementary Figure 4 — Expression profile of miRNAs in HCV-infected Huh7 cells. (A) Ratio of the small RNAs to total expression abundance in Huh7 cells. Each bar represents average expression proportion of more than two replicates at a time point. The right bars at the dashed red line are proportional to the right y-axis. (B) Characteristic of miRNA expression levels in Huh7 cells. The miRNA expression levels are mean of 17 analyzed samples. (C) Histogram of average miRNA expression abundance of 17 samples. (D) The 25 highest expressed miRNAs with > 5,000 RPM in nine HCV-infected Huh7 cells and eight control. The order in expression abundance of the miRNAs is slightly different between infected cells and control. [file Image_4.TIF]

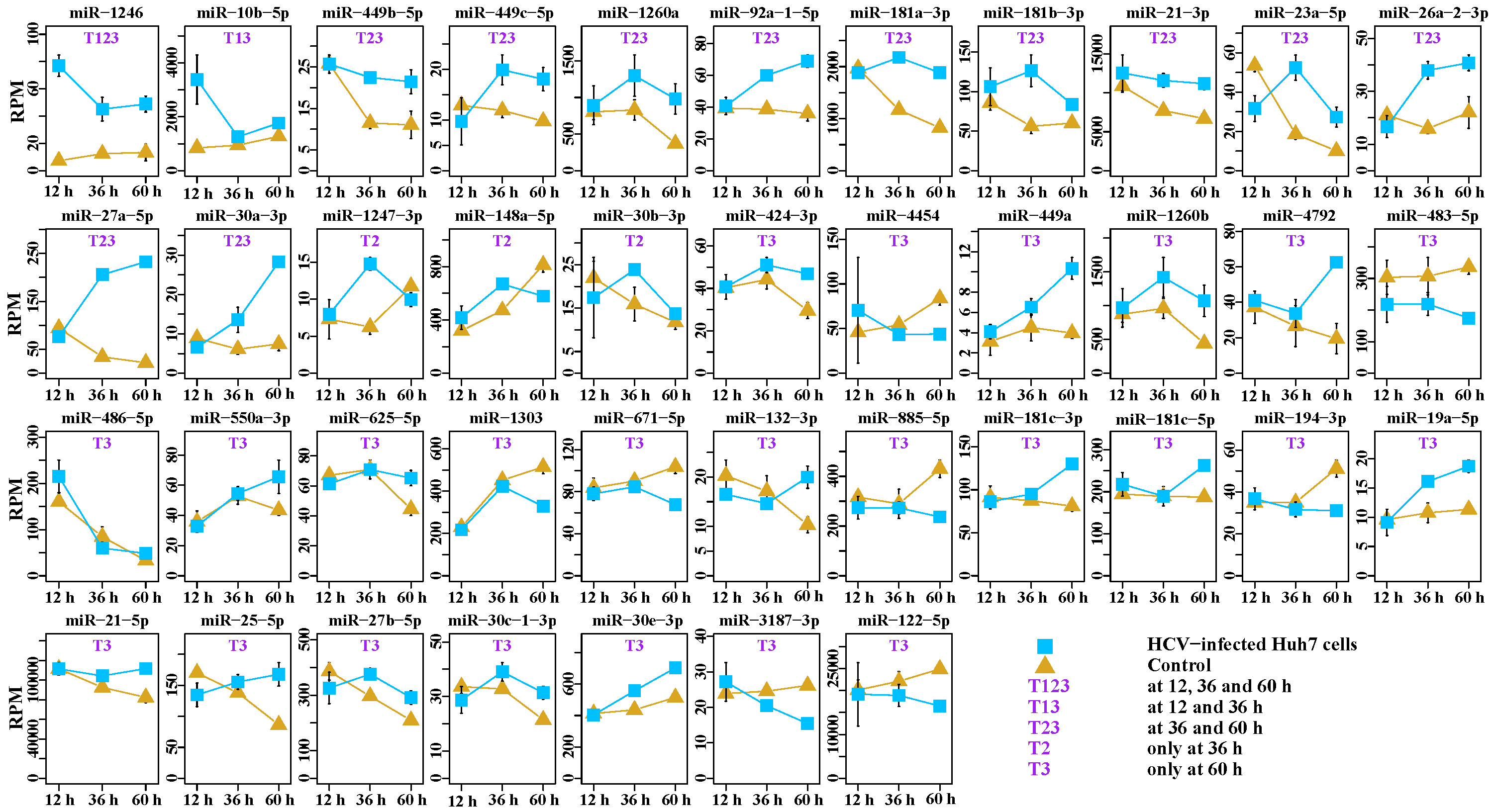

Supplement: Supplementary Figure 5 — miRNA expression variation in HCV-infected Huh7 cells and control over infection time. A total of 42 DE miRNAs at the three time points were presented. Y-axis indicates miRNA expression abundance with reads per million (RPM). The purple-labeled character on each plot shows whether the miRNA is dysregulated at that time, for example, miR-1246 was significantly deregulated at 12, 36, and 60 h, and labeled by T123. The other labels refer to the legend. Data are shown as the mean ± SD. [file Image_5.TIF]

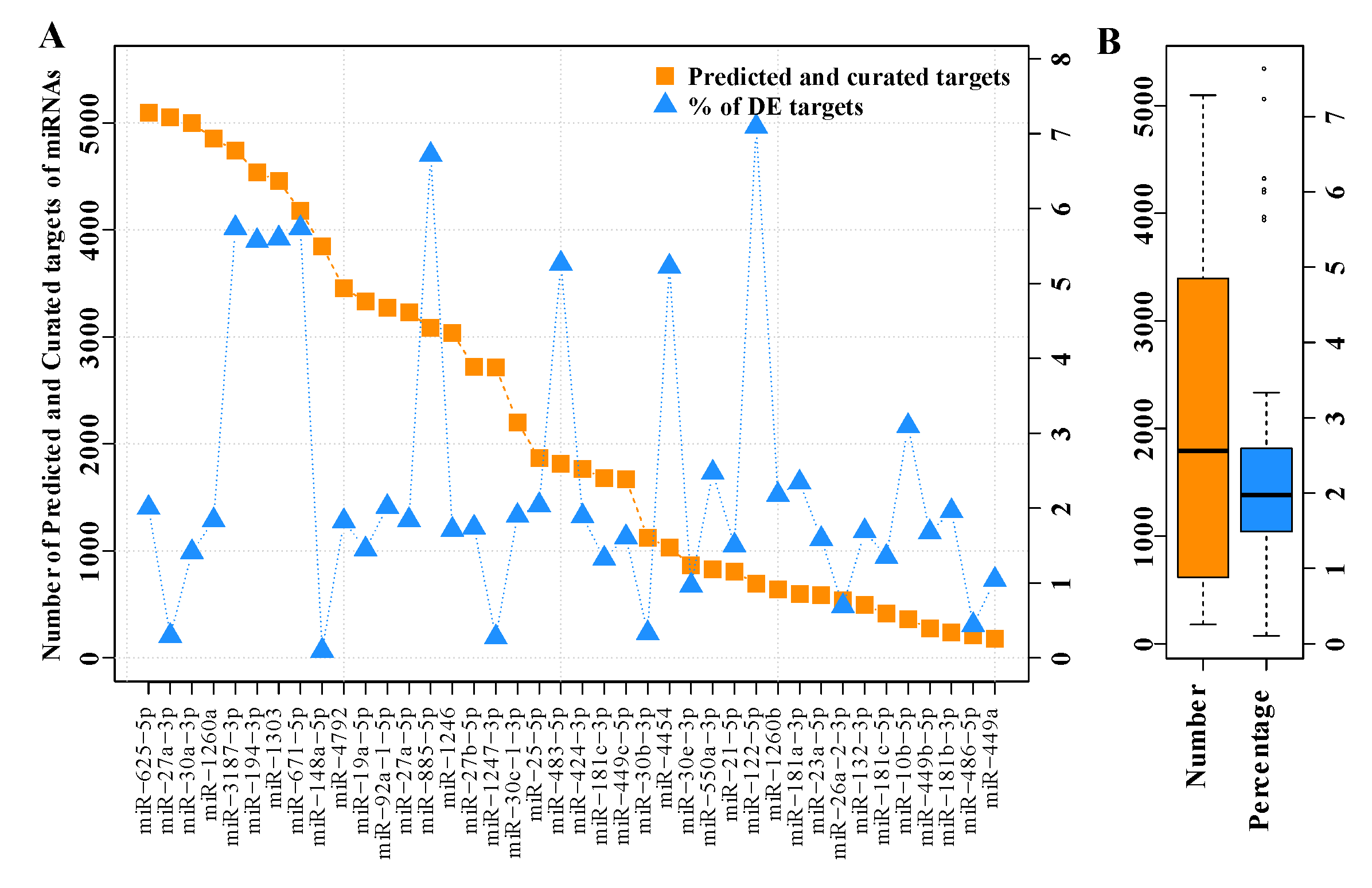

Supplement: Supplementary Figure 6 — Number of and percentage of differentially expressed targets in the integrated targets of the 42 miRNAs. (A) Predicted targets of the differentially expressed miRNAs. Darkorange rectangles indicate number of predicted (TargetScan) and curated targets (miRBase) of the miRNAs. Dodgerblue triangles indicates percentage of the differentially expressed targets in predicted and curated targets and the percentage corresponds to the right y-axis. (B) Boxplot of predicted targets in (A). [file Image_6.TIF]

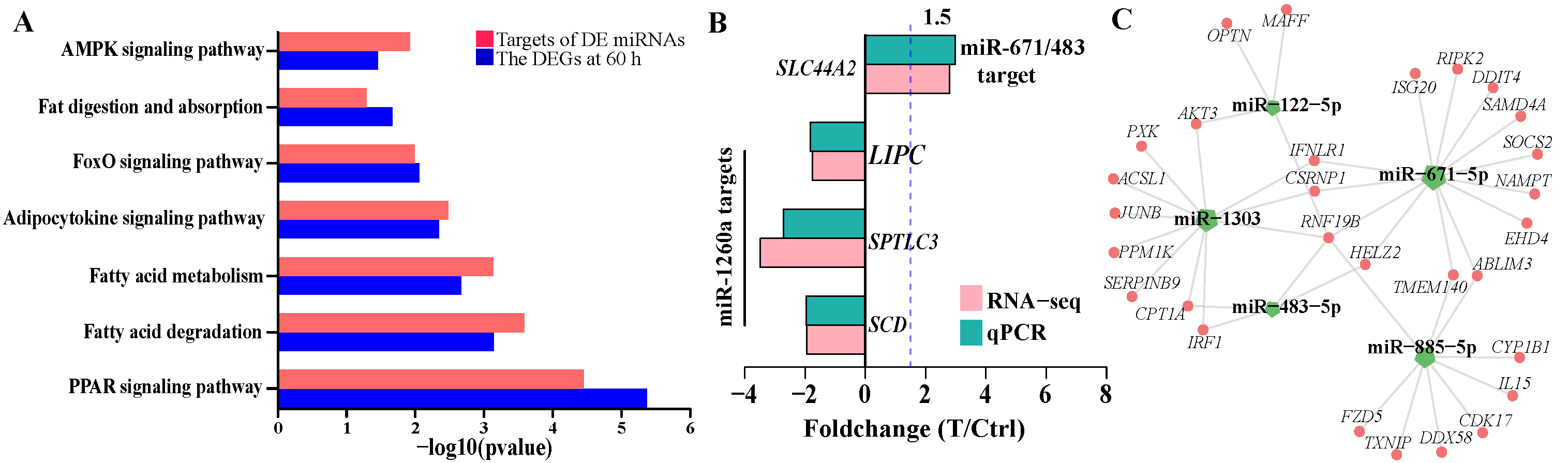

Supplement: Supplementary Figure 7 — Pathways and interferon-stimulated genes regulated by miRNAs. (A) Comparison of the enriched KEGG pathways between all differentially expressed genes and the predicted targets by the differentially expressed miRNAs at 60 h. The pathways involved in lipid metabolisms are exhibited. (B) qPCR validation of gene expression changes targeted by miRNAs. (C) Interferon-stimulated genes regulated by downregulated miRNAs. Each edge between miRNAs and interferon-stimulated gene represents a predictive regulation. [file Image_7.TIF]

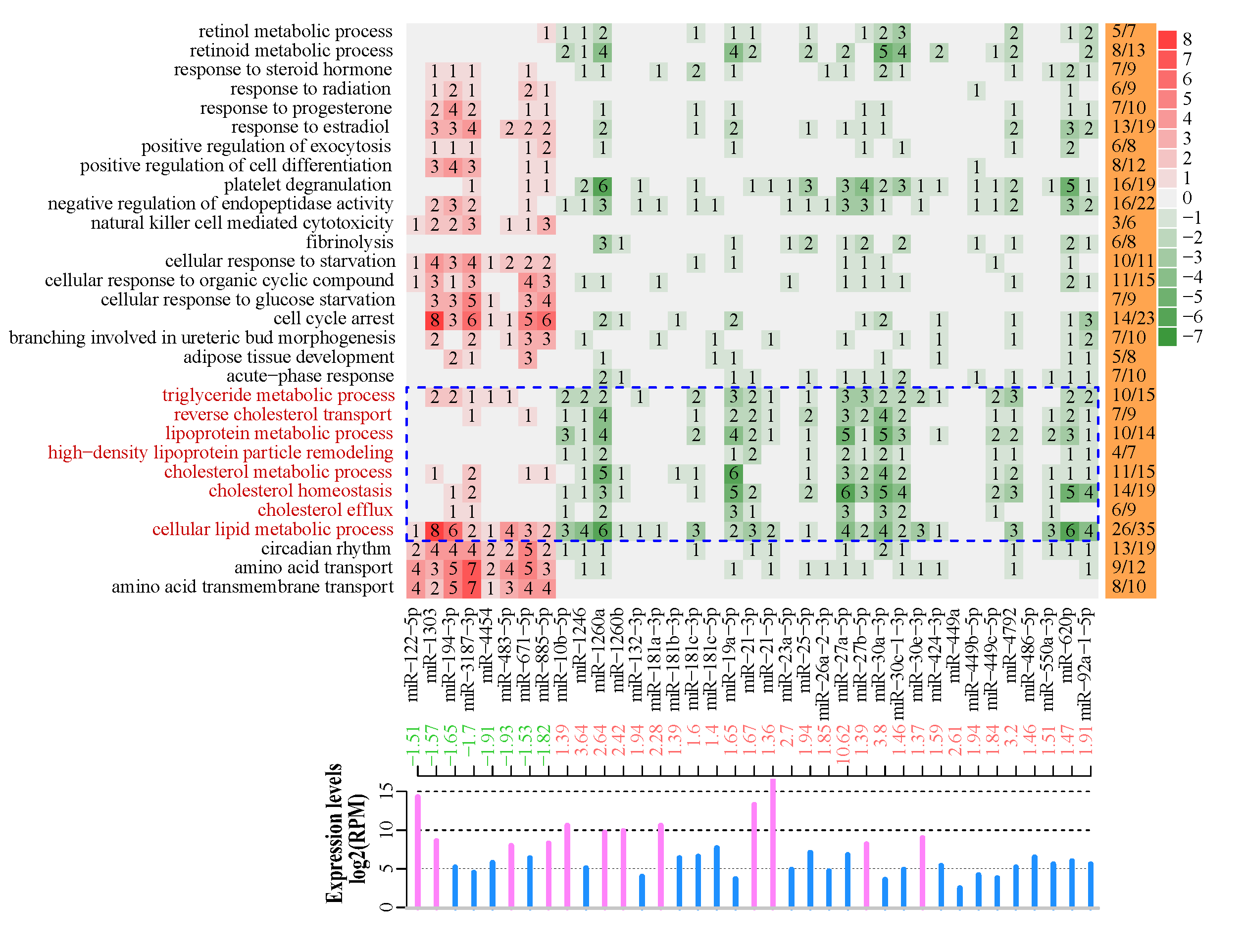

Supplement: Supplementary Figure 8 — Deregulated miRNAs and their putative involvement in the enriched Gene Ontology (GO) biological processes at 60 h. X-axis represents deregulated miRNAs and y-axis indicates enriched GO biological processes. Gene number in one GO biological process regulated by one miRNA is labeled in box. Expression levels and alteration of the miRNAs were presented using bars and colored fold changes, respectively. The Number of differentially expressed genes targeted by miRNAs in a GO biological process and all differentially expressed genes belonging to that GO biological processes were highlighted by orange at the right. [file Image_8.TIF]
